# Evaluating a common semi-mechanistic mathematical model of gene-regulatory networks

**DOI:** 10.1186/1752-0509-9-S5-S2

**Published:** 2015-09-01

**Authors:** Alexandru Mizeranschi, Huiru Zheng, Paul Thompson, Werner Dubitzky

**Affiliations:** 1 University of Ulster, Coleraine, UK; 2 University of Ulster, Jordanstown, UK

**Keywords:** Modeling and simulation, Gene-regulatory networks, Automated model inference

## Abstract

Modeling and simulation of gene-regulatory networks (GRNs) has become an important aspect of modern systems biology investigations into mechanisms underlying gene regulation. A key challenge in this area is the automated inference (reverse-engineering) of *dynamic, mechanistic GRN models *from gene expression time-course data. Common mathematical formalisms for representing such models capture two aspects simultaneously within a single parameter: (1) Whether or not a gene is regulated, and if so, the *type of regulator *(activator or repressor), and (2) the *strength of influence *of the regulator (if any) on the target or effector gene. To accommodate both roles, "generous" boundaries or limits for possible values of this parameter are commonly allowed in the reverse-engineering process. This approach has several important drawbacks. First, in the absence of good guidelines, there is no consensus on what limits are reasonable. Second, because the limits may vary greatly among different reverse-engineering experiments, the concrete values obtained for the models may differ considerably, and thus it is difficult to compare models. Third, if high values are chosen as limits, the search space of the model inference process becomes very large, adding unnecessary computational load to the already complex reverse-engineering process. In this study, we demonstrate that restricting the limits to the [−1, +1] interval is sufficient to represent the essential features of GRN systems and offers a reduction of the search space without loss of quality in the resulting models. To show this, we have carried out reverse-engineering studies on data generated from artificial and experimentally determined from real GRN systems.

## Introduction

Systems biology refers to the quantitative analysis of the dynamic interactions among multiple components of a biological system and aims to understand the characteristics of a system as a whole [1,2]. It involves the development and application of system-theoretic concepts for the study of complex biological systems through iteration over mathematical modeling, computational simulation and biological experimentation. The regulation of genes and their products is at the heart of a systems view of complex biological processes. Hence, the modeling and simulation of *gene-regulation networks *(*GRNs*) is becoming an area of growing interest in systems biology research [3]. For instance, understanding gene-regulatory processes in the context of diseases is increasingly important for therapeutic development. Cells regulate the expression of their genes to create functional gene products (RNA, proteins) from the information stored in genes (DNA). Gene regulation is a complex process involving the transcription of genetic information from DNA to RNA, the translation of RNA information to make protein, and the post-translational modification of proteins. Gene regulation is essential for life as it allows an organism to respond to changes in the environment by making the required amount of the right type of protein when needed. Developing *quantitative models of gene regulation *is essential to guide our understanding of complex gene-regulatory processes and systems. The approach considered in this study concentrates on a conceptualization of GRNs that ignores intricate intermediate biological processes of cellular gene regulation, such as splicing, capping, translation, binding and unbinding [4].

As the amount of gene expression data is growing, researchers are becoming increasingly interested in the *automated inference *or *reverse-engineering *of quantitative dynamic, mechanistic gene-regulatory network *models *from gene expression time-course data [5,4,1-9]. The quality of such reverse-engineered GRN models is determined mainly by two factors:

The quality of a GRN model depends on two factors: the model's *explanatory power *(or model completeness) and the model's *predictive power *(or model correctness).

A model's explanatory power depends on how well the elements of a mathematical model specification correspond to the salient features of the modelled system. Thus, the explanatory power depends crucially on the concrete *form *of the chosen mathematical model. This is a decision made by the modeler at the start of a modeling process, hence it is related to the *modeling error*. A poor choice at this stage means low explanatory power and high modeling error. Even if an adequate model form is chosen, we can still end up with a model that has a low explanatory power. This can happen if we create a model with parameter values that are unrealistic, i.e. are not in good agreement with the corresponding system features.

A model's predictive power is estimated by simulating the system's response to the initial condition captured in an independent validation dataset [10]. The greater the deviation (error) between the response time courses predicted by the model and the actual time courses in the validation data, the lower the predictive power of the model.

The quality of the model inference or model reverse-engineering algorithm is referred to as *inferential power*. The inferential power depends on the quality of the inferred GRN model. The higher the quality of the inferred model (in terms of explanatory and predictive power), the higher the inferential power of the algorithm. Reverse-engineering of GRN models from data is a highly compute-intensive process, hence another crucial aspect in deciding the quality of a reverse-engineering algorithm is the computational performance of its implementation.

Reverse-engineering GRN models with highly accurate structure accuracy and predictive performance is a long-standing problem [4]. Currently, some of the main challenges in reverse-engineering of more accurate and reliable GRN models include:

- A lack of sufficient amounts of gene expression time-course data. While the number of sampling points is important, far more important is to have multiple stimulus-response data sets from the same system [5]. This is a challenging requirement for current experimental practice.

- A lack of reverse-engineering algorithms and methods that are able to incorporate existing biological knowledge effectively.

**Figure 1:**
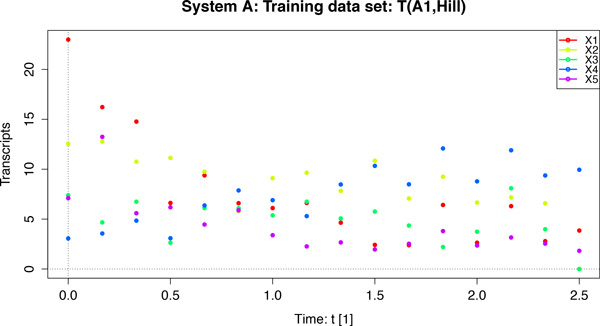
**Training data set with Gaussian noise added**.

In this study, we focus on an intricate aspect of the GRN modelling and simulation that links predictive and inferential power. Based on two common mathematical GRN model formalisms, we analyze the effect that the "structure parameter" of these formalisms has on the quality of the inferred models. In order to assess this, we have performed various reverse-engineering experiments on synthetic data based on three different 5-gene GRN systems, as well as on data obtained from an 11-gene yeast cell-cycle system [11]. This study is *not *about presenting a new method, but about analyzing a particular property of common GRN model formalisms in the context automated of GRN model inference. To account for systematic bias and random variation, we have designed our experiment based on 4 different GRN systems (3 artificial, 1 biological). For the artificial systems, we have generated multiple data sets under the various realistic noise conditions to mimic real data as closely as possible. Figure 1 shows a training data set created from artificial system A with the Hill rate law (Eq. 1).

The main contribution of this study is to provide insight into the behavior of the structure parameter of commonly used GRN model formalisms and guidelines on how to deal with this parameter in similar optimization-based reverse-engineering procedures. Thus, the contribution of this study is not about a new method for GRN model inference, but a better understanding of the characteristics of existing formalisms in the context of automated GRN model inference procedures. We believe this is an important contribution, as it will help scientists to understand better the relationship between formalisms used to represent GRN models and automated procedures that generate such models from gene expression data.

The remainder of this paper is organized as follows: We first present two common semi-mechanistic mathematical models used to represent GRN systems, and an algorithm to automatically infer (reverse-engineer) such models from gene-expression time-course data. We then describe the main hypothesis underlying this study, the data (synthetic and biological) we used, and the basic experimental design and setup of the computational experiments we performed. This is followed by a section presenting the results of the experiments and their discussion and interpretation. First, we present and discuss training and validation errors obtained from the 192 GRN models derived from the 24 training data sets generated from 3 synthetic 5-gene GRN systems. Then we present and discuss the training/validation errors from the 11-gene GRN models we inferred from a yeast data set. Finally, in the Conclusions section, we reflect on the results of this study in the broader context of inferring reliable GRN models from time-series gene expression data.

## Methods

### Rate law

The main assumption behind automated GRN model inference from timecourse gene expression data is that such data contains sufficient information to generate models that capture the essential mechanistic characteristics of the underlying biological GRN system. A common strategy for modeling and simulating dynamic GRNs is based on nonlinear *ordinary differential equations *(*ODEs*) that are derived from standard mass-balance kinetic rate laws [2]. The ODEs in a GRN model relate changes in gene transcripts concentration to each other (and possibly to an external perturbations). Such models consist of one ODE for each gene in the GRN, where each equation describes the transcription rate of the gene as a function of the other genes (and of the external perturbations). The parameters of the equations have to be inferred from the expression time-course data. ODE GRN models are similar to metabolic models that are formulated based on enzyme kinetics, where each rate law approximates a series of elementary chemical steps. Here, the rate laws are one level of complexity above that and represent a *series *of enzymatic steps. Because these rate laws combine mechanistic details into a small set of model parameters, they are sometimes referred to as "lumped" or "semimechanistic" models. In a sense, these models are neither fully mechanistic nor purely phenomenological.

This study is based on two commonly used rate law formulations: the *Hill *rate law [2,12], defined by Eq. 1, and the *artificial neural network *(*ANN*) rate law [13], defined by Eq. 2.

(1) $$\begin{array}{c} \frac{dx_{i}}{dt}={\hat{\alpha }}_{i}\overset{n}{\underset{j}{\sum }}\left|\omega_{ij}\right|r_{i}\left(x_{i}\right)-\beta_{i}x_{i},\;with\\ r_{i}\left(x_{j}\right)=\left\{\begin{array}{cc} {x_{j}}^{n_{ij}}/\left({x_{j}}^{n_{ij}}+{\gamma_{i}}^{n_{ij}}\right) & if\omega_{ij}>0\\ 1/(1+(x_{j}/\gamma_{i})n_{ij} & if\omega_{ij}<0 \end{array}\right) \end{array}$$

(2) $$\frac{dx_{i}}{dt}={\hat{\alpha }}_{i}\frac{1}{1+\text{exp}\left(\gamma_{i}-\overset{n}{\underset{j}{\sum }}\omega_{ij}x_{j}\right)}-\beta_{i}x_{i}$$

**- ***x_i_, x_j _*∈ {*x*_1_*, x*_2_*, ..., x_n_*}: Time-dependent *transcript concentration *of gene *i *and *j*, respectively, where *n *is the total number of genes in the GRN system;

**- ***dx_i_/dt *∈ ℝ: *Total rate of x_i _change *at time *t*;

**- ${\hat{\alpha }}_{i}\in {\mathbb{R}}^{+}:$***Maximal synthesis rate *of transcript *x_i_*;

**- ***ω_ij _*∈ ℝ: *Type *of synthesis regulation of transcript *x_i _*by *x_j_*, such that

*ω_ij _>*0: *Synthesis activation *of *x_i _*by *x_j_*;

*ω_ij _<*0: *Synthesis repression *of *x_i _*by *x_j_*;

*ω_ij _*= 0: *No synthesis regulation *of *x_i _*by *x_j_*.

**-$|\omega_{ij}|\;\in \;{\mathbb{R}}_{0}^{+}:$***Relative weight *of synthesis-regulatory influence of *x_j _*on *x_i_*;

**- ***γ_i_*: *Activation/repression coefficient *of gene *i*; *γ_i _*∈ ℝ for ANN, and

*γ_i _*∈ ℝ^+ ^for Hill;

**- ***n_ij _*∈ ℝ^+^: *Hill coefficient *controlling the steepness of the sigmoidal regulation function; and

**- ***β_i _*∈ ℝ^+^: *Degradation rate constant *modulating the degradation rate of *x_i_*.

Both rate laws have been shown to represent essential characteristics of biological processes [2,8,12-15]. They capture a maximal synthesis rate (${\hat{\alpha }}_{i}$), sigmoidal (saturable) kinetics, and an activation/repression threshold (*γ_i_*). For *nij <*1, the Hill rate law represents Michaelis-Menten kinetics. The rate law versions shown in Eqs. 1 and 2 assume additive input processing and a linear transcript degradation rate (*β_i_x_i_*) that depends only on the concentration of the target gene's product. These assumptions are not set in stone; the rate laws may be adapted to capture multiplicative input processing and a non-linear degradation rate which may depend on various influences. Variations that capture basal transcript synthesis and input delays are also possible [8,2].

Like in other comparable GRN rate laws (e.g. the synergistic-system [16]), the omega parameter (*ω_ij_*) represents two distinct biological concepts simultaneously; a discrete as well as a continuous concept. On one hand, it defines the nature or *type of synthesis regulation *between two genes *i *and *j*: if *ω_ij _>*0, then gene *j activates *synthesis of transcript *x_i_*, if *ω_ij _<*0, then gene *j represses x_i _*synthesis, and if *ω_ij _*= 0, then gene *j *does not regulate transcript *x_i _*at all. Hence, the totality of all *ω_ij _*parameters determines the transcript *synthesis*-regulatory network structure of the GRN. On the other hand, the quantity *|ω_ij _| *defines the *strength *or influence of a regulator gene *j *on its target or effector gene *i*. When we employ automated reverse-engineering of GRN models from time-course gene expression data with algorithms like the one illustrated in Algorithm 1, the dual role of *ω_ij _*and its discrete-continuous interpretation has important consequences.

First, because *ω_ij _*needs to be coded as a real number (*ω_ij _*∈ ℝ), the chances of a typical parameter estimation algorithm to find *ω_ij _*= 0 are very small. Thus, reverse-engineering algorithms like the one discussed below have a tendency to infer only non-zero values for *|ω_ij _|*, representing *fully *connected network structures. Fully connected GRN network structures are at best very difficult to interpret biologically, at worst meaningless.

Second, because typical GRN model formalisms like the ones in Eqs. 1 and 2 contain additional parameters to represent other quantitative aspects of GRN systems, reverse-engineering algorithms tend to "balance" the quantitative values of all parameters. This means that only the *relative *quantities *|ω_ij _| *are important, not their absolute values! It is important to understand this property, as this lies at the heart of this study.

Third, in the absence of a clear understanding of the effect *ω_ij _*has in the inference process, there is a danger that modelers choose large omega intervals in their algorithms. This, of course, adds additional computational load because it increases the size of the search or solution space.

### Inference algorithm

Once one has chosen a rate law or model formalism to represent a GRN, one needs to determine the *concrete values *of the model's parameters - the parameters that describe the network structure, and the parameters that represent other aspects of the modeled GRN system. If these parameters are not known, they are typically inferred by reverse-engineering or parameter estimation algorithms like the one defined by Algorithm 1.

Given stimulus-response gene expression time-course data, *D*, and a particular model formulation, *M*, Algorithm 1 determines concrete parameter values. The algorithm iterates over three main steps:

1. An *optimizer *algorithm that generates candidate model parameter values by attempting to minimize the training error, *E*.

2. An *ODE solver *component that numerically integrates the model equations using the initial values of the time series in the training data set, *D*.

**Input**: *M ← *Model equations; *L ← *Parameter limits; *G ← *Network topology; *D ← *Training data; *ε ← *Error threshold

**Output**: *P ← *Parameter values; *E ← *Training error;

(* Initialize and process: *)

*S ← *Simulation data (* Initialize *)

*E ← ∞ *(* Initialize *)

repeat

*P ← Optimize*(*L, E*) (* Parameter values *)

*S ← SolveODE*(*M, P, D*) (* Solve model *)

*E ← Error*(*S, D*) (* Determine error *)

**until ***E < ε *;

**Algorithm 1: **Basic reverse-engineering algorithm. The network topology, *G*, is an optional input. In this study, we experiment with known network topology only.

3. A component that computes the *simulation error, E*, based on the gene expression time-course data in the training data set, *D*, and the predicted or simulated data, *S*, determined by the ODE solver.

**Figure 2:**
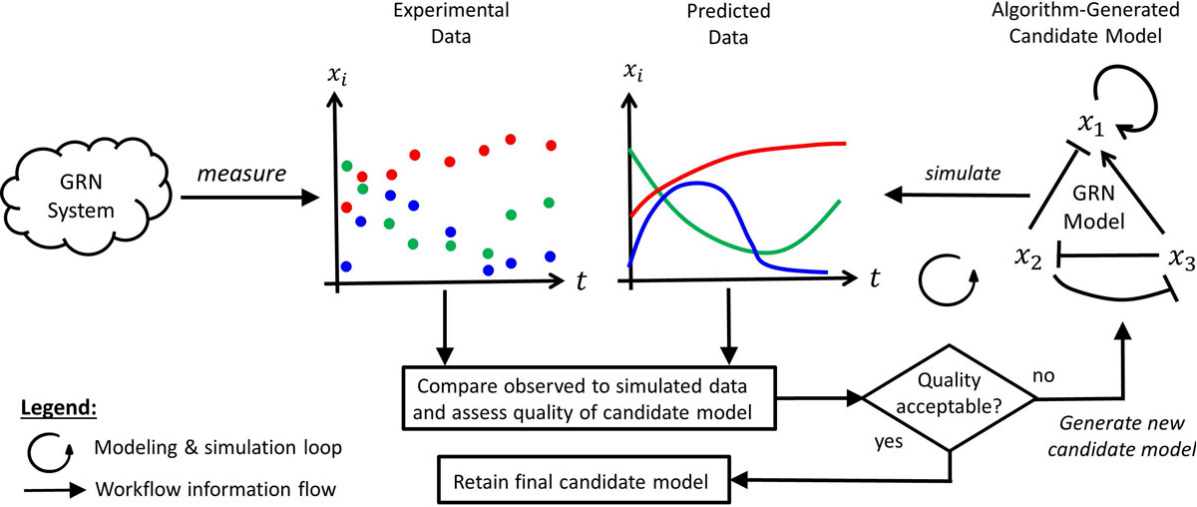
**GRN model reverse-engineering workflow**. The modeling and simulation loop keeps generating models which predict data until the quality of a candidate is deemed acceptable.

GRN modeling and simulation software tools implement various features that realize the elements listed above. The diagram in Figure 2 depicts the basic components and "workflow" of a typical GRN model reverse-engineering algorithm. The cloud shape on the left represents the GRN system under study. In this simplified example, the GRN system has 3 genes corresponding to the model variables *x*_1_*, x*_2_*, x*_3_, respectively. The series of dots labeled Experimental Data illustrates the three gene-expression time-series that have been experimentally obtained from the GRN system over 8 consecutive time points. We refer to this as the *training dataset*. The right part of Figure 2 shows the main reverse-engineering loop. The network shape on the right (labeled GRN Model) is a graphical depiction of a algorithm-generated concrete candidate model with gene-regulatory interaction links between the three genes of the system. The algorithm simulates the system based on the candidate model and the initial condition of the dataset (arrow labeled simulate). The simulation produces a predicted or simulated dataset (curves labeled Predicted Data). The experimental and predicted data are then compared (diamond shape) to assess the quality of the candidate model. If the quality is deemed acceptable, the candidate model is retained as the final candidate model. The final candidate model is still subject to validation on independent data (this is not depicted in the diagram).

The simulation of the predicted time series depicted in Figure 2 involves the numeric integration of the model equations. In terms of computational effort, the ODE solver step accounts for approximately for 80% of the total computing time of Algorithm 1. The reverse-engineering process terminates, when the training error drops below a pre-defined error threshold, or when a maximum number of model evaluations is reached.

For this study we have employed the GRN modeling and simulation tool MultGrain/MAPPER. The tool was developed as a part of the European FP7 project Multiscale Applications on European e-Infrastructures (MAPPER) [17]. The goal of MAPPER was to develop a general framework and technology facilitating the development, deployment and execution of *distributed *multiscale modeling and simulation applications [18,19]. Based on tools and services developed in the MAPPER project, MultiGrain/MAPPER realizes the GRN model reverse-engineering process (Figure 2) based on a multi-swarm particle swarm optimization algorithm.

In order to estimate the model parameters, we used the our own implementation of a multi-swarm *particle swarm optimization *[20] (*PSO*) algorithm. PSO is a population-based meta-heuristic inspired by the flocking, schooling or swarming behavior of animals. Two main advantages of this method include that it optimizes continuous variables and it has the ability to avoid getting stuck in local minima by using a multi-swarm approach which successively swaps particles across each swarm after a fixed number of iterations in order to increase the "genetic" diversity of the overall swarm. The PSO parameters were set according to the guidelines of Pedersen et al. [21], who performed a meta-analysis of the PSO algorithm, testing its performance for a wide range of parameter values.

## Hypothesis, data and experiments

The "fitness landscape" that the reverse-engineering Algorithm 1 is allowed to explore is defined by the value ranges of the model parameter intervals. The basic meaningful ranges of the GRN model parameters in Eqs. 1 and 2 are specified below the equations. In order to limit the computational effort required to estimate the parameters, practical value ranges are typically much smaller than those shown.

In this study we have tested the following hypothesis: *ω_ij _*∈ [−1, +1] is a sufficiently large permissible range for the important *ω_ij _*parameter values, because it is expressive enough

1. To encode the three regulatory interaction possibilities (synthesis activation, synthesis repression, no synthesis regulation) between two genes *i *and *j*, and

2. To represent the strength of the regulatory influence of gene *j *on *i*. As we have discussed, only the *relative *values of *|ω_ij _| *are relevant, because of the way the *ω_ij _*parameters interact with one another and the other model parameters of the model Eqs. 1 and 2.

To test this hypothesis, we have conducted a number of experiments on data obtained from artificial and real GRN systems.

**Figure 3:**
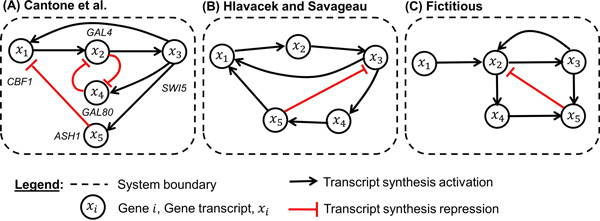
**Artificial 5-gene GRN systems**.

We have created three 5-gene GRN systems (Figure 3): **System A **represents a yeast GRN with five synthesis activating and three synthesis repressing influences [8]. **Systems B **and **C **have six activating influences and one repressing influence (B is modeled on Hlavacek and Savageau [22] and C is a purely fictitious network structure with realistic network features).

For each of the three systems, we have created 4 training and 4 validation data sets with the Hill (Eq. 1) and 4 training and 4 validation data sets with the ANN (Eq. 2) rate law, respectively (Table 1). So in total we created 24 training and 24 validation data sets (the validation sets were created using different initial conditions). The 4 variants per system are distinguished by the encoding of the *ω_ij _*values used to represent the GRN structure. While the sign and zero-values of the *ω_ij _*values are identical across the four variants per system, we have varied the quantity of *ω_ij _*as follows. For *Version 1 *we used only *ω_ij _*∈ {−1, 0, +1}, i.e. *ω_ij _*= *−*1 for synthesis repression, *ω_ij _*= +1 for syn-thesis activation, and *ω_ij _*= 0 for no synthesis regulation. Correspondingly, for *Version 2 *we used only *ω_ij _*∈ {−5, 0, +5}, for *Version 3 ω_ij _*∈ {−10, 0, +10}, and for *Version 4 ω_ij _*∈ {−20, 0, +20}. For example, in Table 1 "V(B5,Hill)" refers to the validation data set from system B created with *ω_ij _*= −5 representing a synthesis repression regulator, *ω_ij _*= +5, a synthesis activation regulator, and *ω_ij _*= 0 no synthesis regulation.

**Table 1:** Training/validation data sets for each artificial systems A, B and C, with four different *ω_ij _*intervals.

	ANN training data			ANN validation data		
	
ω−value	A	B	C	A	B	C
**{-1, 0, +1}**	T(A1,ANN)	T(B1,ANN)	T(C1,ANN)	V(A1,ANN)	V(B1,ANN)	V(C1,ANN)
**{-5, 0, +5}**	T(A5,ANN)	T(B5,ANN)	T(C5,ANN)	V(A5,ANN)	V(B5,ANN)	V(C5,ANN)
**{-10, 0, +10}**	T(A10,ANN)	T(B10,ANN)	T(C10,ANN)	V(A10,ANN)	V(B10,ANN)	V(C10,ANN)
**{-20, 0, +20}**	T(A20,ANN)	T(B20,ANN)	T(C20,ANN)	V(A20,ANN)	V(B20,ANN)	V(C20,ANN)

	**Hill training data**			**Hill validation data**		
	
	**A**	**B**	**C**	**A**	**B**	**C**

**{-1, 0, +1}**	T(A1,Hill)	T(B1,Hill)	T(C1,Hill)	V(A1,Hill)	V(B1,Hill)	V(C1,Hill)
**{-5, 0, +5}**	T(A5,Hill)	T(B5,Hill)	T(C5,Hill)	V(A5,Hill)	V(B5,Hill)	V(C5,Hill)
**{-10, 0, +10}**	T(A10,Hill)	T(B10,Hill)	T(C10,Hill)	V(A10,Hill)	V(B10,Hill)	V(C10,Hill)
**{-20, 0, +20}**	T(A20,Hill)	T(B20,Hill)	T(C20,Hill)	V(A20,Hill)	V(B20,Hill)	V(C20,Hill)

All of the synthetic data sets consist of measurements over 16 consecutive time points. After the data sets were created, we added zero-mean Gaussian noise (values are drawn from a normal random variable with a mean of zero and a variance of 0.15 times the maximum range of all the expression levels) [23].

In addition to the three artificial 5-gene GRN systems, we used two real data sets obtained from 11 **yeast cell cycle **genes [11]. One data set (38 time points) was used for training, and the other (30 time points) for validation. The network structure of this 11-gene yeast cell cycle system consists of 15 activating and 14 repressing influences [24].

To determine the role that the omega parameters play in GRN model inference, we reverse-engineered a total of 192 GRN models from the 24 synthetic training data sets. Each of the 24 training data sets depicted in Table 1 was reverse-engineered 4 times with the Hill (Eq. 1), and 4 times with the ANN (Eq. 2) rate law, with the following interval settings for the omega parameters: *ω_ij _*∈ [−1, +1], *ω_ij _*∈ [−5, +5], *ω_ij _*∈ [−10, +10] and *ω_ij _*∈ [−20, +20]. Notice, in the reverse-engineered *models*, the parameters are free to assume any value within the given interval limits, whereas in the artificial *systems *(Section 3) the same parameters assume only the boundary values of these intervals (for synthesis activation and repression), and zero for no synthesis regulation (hence the first column in Table 1 does not show intervals but sets that contain exactly three elements).

In addition to the 192 GRN models we reverse-engineered from the data generated from our artificial systems, we have reverse-engineered 8 GRN models from the single training data set (Alpha 38) of the yeast cell cycle system using both the Hill and ANN rate laws with the same interval specifications for the omega parameters: *ω_ij _*∈ [*−*1, +1], *ω_ij _*∈ [*−*5, +5], *ω_ij _*∈ [*−*10, +10] and *ω_ij _*∈ [*−*20, +20].

Each of the 192 GRN models from synthetic data was validated against the corresponding independent validation data set, and the each of the 8 models inferred from the yeast cell cycle system was validated against the single independent validation data set (Alpha 30).

## Results and discussion

The training and validation errors (*normalised root mean squared errors*) of our experiments are shown in Tables 2, 3, 4, 5 and 6 below. In the tables $\bar{x}$ and *s *denote the mean error and error standard deviation, respectively, obtained from four reverse-engineering replications per model. Rows in these tables refer to the GRN *systems *from which the data was obtained, and columns to omega intervals used to reverse-engineer the GRN *models*.

**Table 2:** Training errors of models of synthetic systems A, B, C which were created with *ANN *rate law.

System and Code		Training data from synthetic ANN SYSTEM									
				
		ANN MODEL training error				Hill MODEL training error					
				
		[-1,+1]	[-5,+5]	[-10,+10]	[-20,+20]	[-1,+1]	[-5,+5]	[-10,+10]	[-20,+20]	$\bar{x}$	*s*
**A**	**-1, 0, +1**	0.135	0.136	0.135	0.136	0.139	0.140	0.136	0.133	0.136	0.002
**B**		0.161	0.160	0.160	0.160	0.176	0.163	0.159	0.162	0.163	0.006
**C**		0.160	0.160	0.160	0.160	0.158	0.160	0.158	0.166	0.160	0.002

**A**	**-5, 0, +5**	0.137	0.136	0.139	0.137	0.142	0.141	0.136	0.143	0.139	0.003
**B**		0.128	0.126	0.124	0.124	0.139	0.125	0.126	0.124	0.127	0.005
**C**		0.152	0.152	0.152	0.152	0.153	0.155	0.151	0.153	0.152	0.001

**A**	**-10,0,+10**	0.156	0.156	0.156	0.156	**0.186**	**0.160**	**0.158**	**0.148**	0.160	0.011
**B**		0.142	0.142	0.142	0.142	0.149	0.150	0.150	0.139	0.144	0.004
**C**		0.144	0.142	0.142	0.142	0.147	0.150	0.148	0.149	0.146	0.003

**A**	**-20,0,+20**	0.132	0.136	0.136	0.134	0.141	0.137	0.138	0.131	0.136	0.003
**B**		0.135	0.137	0.136	0.136	0.137	0.145	0.145	0.136	0.138	0.004
**C**		*0.131*	*0.130*	*0.130*	*0.130*	*0.132*	*0.130*	*0.130*	*0.131*	0.130	0.001

$\bar{x}$		0.143	0.143	0.143	0.143	0.150	0.146	0.145	0.143	**ALL**	**ALL**

*s*		0.012	0.012	0.012	0.012	0.017	0.012	0.011	0.013	0.144	0.012

**Table 3:** Training errors of models of synthetic systems A, B, C which were created with *Hill *rate law.

System and Code		Training data from synthetic Hill SYSTEM									
				
		ANN MODEL training error				Hill MODEL training error					
				
		[-1,+1]	[-5,+5]	[-10,+10]	[-20,+20]	[-1,+1]	[-5,+5]	[-10,+10]	[-20,+20]	$\bar{x}$	*s*
**A**	**-1, 0, +1**	*0.118*	0.115	0.120	*0.116*	0.126	0.119	0.119	0.137	0.121	0.007
**B**		*0.140*	0.143	0.143	*0.143*	0.147	0.141	0.141	0.138	0.142	0.003
**C**		*0.165*	0.163	0.164	*0.167*	0.163	0.162	0.162	0.163	0.163	0.002

**A**	**-5, 0, +5**	*0.252*	0.254	0.252	*0.252*	0.134	0.133	0.134	0.133	0.193	0.064
**B**		*0.230*	0.247	0.247	*0.247*	0.126	0.126	0.127	0.123	0.184	0.063
**C**		*0.348*	0.348	0.348	*0.348*	0.132	0.132	0.131	0.133	0.240	0.116

**A**	**-10,0,+10**	*0.470*	0.471	0.470	*0.470*	0.153	0.152	0.152	0.153	0.311	0.170
**B**		*0.455*	0.447	0.443	*0.455*	0.125	0.125	0.125	0.124	0.287	0.174
**C**		*0.432*	0.432	0.432	*0.432*	0.126	0.112	0.111	0.112	0.274	0.170

**A**	**-20,0,+20**	*0.586*	0.586	0.586	*0.586*	**0.194**	**0.175**	**0.168**	**0.168**	0.381	0.219
**B**		*0.568*	0.568	0.568	*0.568*	**0.141**	**0.122**	**0.117**	**0.116**	0.346	0.237
**C**		*0.492*	0.492	0.492	*0.492*	**0.194**	**0.140**	**0.124**	**0.123**	0.318	0.187

$\bar{x}$		0.355	0.355	0.355	0.356	0.147	0.136	0.134	0.135	**ALL**	**ALL**

*s*		0.168	0.167	0.166	0.167	0.025	0.019	0.018	0.018	0.247	0.158

**Table 4:** Validation errors of models of synthetic systems A, B, C which were created with *ANN *rate law.

System and Code		Validation data from synthetic ANN SYSTEM									
				
		ANN MODEL validation error				Hill MODEL validation error					
				
		[-1,+1]	[-5,+5]	[-10,+10]	[-20,+20]	[-1,+1]	[-5,+5]	[-10,+10]	[-20,+20]	$\bar{x}$	*s*
**A**	**-1, 0, +1**	0.220	0.199	0.193	0.168	**0.243**	**0.397**	**0.362**	**0.405**	0.273	0.098
**B**		0.168	0.140	0.166	0.164	**0.261**	**0.166**	**0.266**	**0.431**	0.220	0.097
**C**		0.161	0.141	0.148	0.161	0.291	0.353	0.300	0.260	0.227	0.083

**A**	**-5, 0, +5**	0.228	0.217	0.216	0.199	0.304	0.382	0.342	0.463	0.294	0.096
**B**		**0.144**	**0.292**	**0.133**	**0.134**	**0.163**	**0.177**	**0.147**	**0.379**	0.196	0.090
**C**		0.160	0.150	0.141	0.140	0.244	0.254	0.209	0.312	0.201	0.064

**A**	**-10,0,+10**	0.163	0.159	0.155	0.198	0.329	0.298	0.255	0.375	0.242	0.086
**B**		0.198	0.192	0.187	0.188	**0.227**	**0.194**	**0.198**	**0.432**	0.227	0.084
**C**		0.144	0.167	0.159	0.181	0.236	0.277	0.265	0.369	0.225	0.077

**A**	**-20,0,+20**	0.183	0.209	0.216	0.213	0.313	0.355	0.404	0.426	0.290	0.097
**B**		0.185	0.174	0.174	0.172	**0.178**	**0.424**	**0.264**	**0.212**	0.223	0.087
**C**		0.243	0.186	0.233	0.183	**0.303**	**0.262**	**0.245**	**0.418**	0.259	0.075

$\bar{x}$		0.183	0.186	0.177	0.175	0.258	0.295	0.271	0.374	**ALL**	**ALL**

*s*		0.033	0.042	0.032	0.024	0.053	0.088	0.072	0.076	0.240	0.087

**Table 5:** Validation errors of models of synthetic systems A, B, C which were created with *Hill *rate law.

System and Code		Validation data from synthetic Hill SYSTEM									
				
		ANN MODEL validation error				Hill MODEL validation error					
				
		[-1,+1]	[-5,+5]	[-10,+10]	[-20,+20]	[-1,+1]	[-5,+5]	[-10,+10]	[-20,+20]	$\bar{x}$	*s*
**A**	**-1, 0, +1**	0.163	0.240	0.187	0.223	*0.215*	0.203	0.190	*0.212*	0.204	0.024
**B**		0.173	0.173	0.174	0.171	*0.189*	0.185	0.195	*0.184*	0.180	0.009
**C**		0.259	0.255	0.228	0.193	*0.258*	0.189	0.263	*0.224*	0.234	0.030

**A**	**-5, 0, +5**	0.398	0.403	0.403	0.403	*0.201*	0.209	0.203	*0.198*	0.302	0.106
**B**		0.292	0.342	0.342	0.298	*0.132*	0.132	0.133	*0.209*	0.235	0.094
**C**		0.386	0.434	0.337	0.435	*0.148*	0.145	0.148	*0.144*	0.272	0.138

**A**	**-10,0,+10**	0.483	0.481	0.507	0.482	*0.163*	0.164	0.169	*0.171*	0.327	0.172
**B**		0.426	0.423	0.418	0.427	*0.132*	0.133	0.132	*0.134*	0.278	0.156
**C**		*0.516*	*0.556*	*0.488*	*0.556*	*0.165*	0.153	0.153	*0.152*	0.343	0.201

**A**	**-20,0,+20**	0.553	0.553	0.552	0.552	*0.185*	0.167	0.160	*0.161*	0.361	0.206
**B**		0.552	0.552	0.552	0.552	*0.146*	*0.135*	*0.142*	*0.143*	0.347	0.219
**C**		0.535	0.563	0.563	0.533	*0.194*	0.138	0.131	*0.134*	0.349	0.215

$\bar{x}$		0.395	0.415	0.396	0.402	0.177	0.163	0.168	0.172	**ALL**	**ALL**

*s*		0.143	0.137	0.143	0.146	0.037	0.028	0.039	0.032	0.286	0.153

**Table 6:** Training and validation errors of cell cycle models.

System	Traing and validation data from Cell Cycle SYSTEM									
**Cell Cycle (alpha 38)**	**Training error ANN MODEL**				**Training error Hill MODEL**					

	**[-1,+1]**	**[-5,+5]**	**[-10,+10]**	**[-20,+20]**	**[-1,+1]**	**[-5,+5]**	**[-10,+10]**	**[-20,+20]**	$\bar{x}$	***s***
			
	0.110	0.111	0.109	0.114	0.110	0.110	0.113	0.113	0.111	0.002

**Cell Cycle (alpha 30)**	**Validation error ANN MODEL**				**Validation error Hill MODEL**				$\bar{x}$	***s***

	**[-1,+1]**	**[-5,+5]**	**[-10,+10]**	**[-20,+20]**	**[-1,+1]**	**[-5,+5]**	**[-10,+10]**	**[-20,+20]**		
			
	0.220	0.416	0.725	0.213	0.201	0.195	0.214	0.214	0.300	0.187

### Training errors synthetic systems

First, we consider the *training errors *of the GRN models derived from the synthetic GRN systems in Tables 2 and 3. The training error's means and standard deviations are shown in the bottom-right corner of the tables.

The list below summarizes the *average *of the means and the standard deviations of the *training errors *for the 4 sets of models across the four omega intervals used to reverse-engineer the models. These are the averages obtained from the sets of 4 mean training error values in the second row from the bottom of Tables 2 and 3. For example, the the average mean of 0.1427 and average standard deviation 0.0001 for the ANN-ANN system/model model configuration (first four main columns in Table 2) is obtained from the sets of four values at the bottom of these columns. We use "*S*(*X*) *→ M *(*X*): average mean error *± *average standard deviation" to denote the system/model configuration and the associated error data; *X *denotes the rate law used to create the system *S *and infer the model *M*, respectively.

**- **Training: *S*(*ANN*) *→ M *(*ANN*): 0.1427 *± *0.0001.

**- **Training: *S*(*ANN*) *→ M *(*Hill*): 0.1459 *± *0.0023.

**- **Training: *S*(*Hill*) *→ M *(*ANN*): 0.3554 *± *0.0009.

**- **Training: *S*(*Hill*) *→ M *(*Hill*): 0.1381 *± *0.0035.

From the average mean training errors, we notice that both sets of Hill models have an average mean training error close to 0.14. This is comparable to average mean training error of the ANN model obtained from the ANN system's data. However, the mean training error (0.3554) of the ANN model obtained from the ANN system's data is more than twice that value. Since the ANN rate law (Eq. 2) has fewer parameters than the Hill rate law (Eq. 1), and hence a smaller degree of freedom, it is harder for the ANN models to fit data obtained from Hill systems. Hill models, on the other hand, can adapt easier to data generated from ANN systems.

While above observations are interesting, the most important information in the context of our investigation relates to the groups of 4 error values for a given system/model combination (e.g. 4 values highlighted in bold font in Table 2), as well as to entire columns of error values (e.g. blocks of errors in italic font in Table 3). Tables 2 and 3 highlight four horizontal groups of 4 training errors in bold; these groups have a standard deviation higher than 0.010. If anything, one would expect the errors to get smaller for larger omega intervals (from left to right), because larger omega intervals relate to a larger solution space. However, in most cases such a pattern is not observed. Indeed, even for the training errors in the bottom three rows in both tables (these were obtained from data of the three systems with large omega values: *−*20 and +20 for repression and activation, respectively), we cannot find a general improvement of training error for increasing omega intervals. For example, in Table 2 the two horizontal groups of 4 training errors highlighted in italic font do not show a clear pattern of decreasing training errors.

Furthermore, when we look at the profiles of the training errors in the columns of both tables, we notice a good pair-wise similarity of training errors (at least within the four columns relating to the same system/model combination). This is illustrated by two columns highlighted in italic font in Table 3. This means that models inferred with different omega intervals show similar training errors for corresponding data sets. There does not seem to be an advantage of using larger omega intervals in the reverse-engineering process.

### Validation errors synthetic systems

The *validation error *of the inferred models characterizes the predictive power of the models. Tables 4 and 5 show the validation errors of the models inferred from the data of the synthetic systems depicted in Figure 3. The mean validation error of all 192 models inferred from the synthetic systems' data is 0.263 with a standard deviation of 0.127 (not shown in tables). So the mean validation error across all models is ca. 34% higher than the mean training error. The variation of the validation errors is similar to that of the training errors (Tables 2 and 3).

The list below summarizes the average of the means and the standard deviations of the *validation errors *of the four sets of models across the four omega intervals used to reverse-engineer the models.

**- **Validation: *S*(*ANN*) *→ M *(*ANN*): 0.1801 *± *0.0076.

**- **Validation: *S*(*ANN*) *→ M *(*Hill*): 0.2994 *± *0.0146.

**- **Validation: *S*(*Hill*) *→ M *(*ANN*): 0.4019 *± *0.0039.

**- **Validation: *S*(*Hill*) *→ M *(*Hill*): 0.1701 *± *0.0050.

The average mean validation errors are consistent with the averages for the mean training errors, in that, the ANN models' predictive performance on the Hill system's data is much poorer than that of the other three models. In fact, the validation errors reveal that inferring models from data that was obtained from systems that were created with the same rate law (as the model), constitutes a considerable bias. The average mean errors for ANN models obtained from ANN system data, and for Hill models from Hill system data are quite low and similar. However, with mixed configurations (different rate law for system and model), we get much higher average mean validation errors. This relates to the important but frequently ignored issue of the *modeling error*. The modeling error is due to the fundamental imperfections that arise when we make abstractions of reality in the form of mathematical or computational models. A model, any model, is by definition an approximation of reality [25]. The modeling error quantifies how well the abstraction approximates reality. Conceptualizing a complex phenomena such as GRN systems as a mathematical or computational model is a relatively new modeling abstraction. More research is required to understand how to assess the modeling error in such approaches.

Looking at the data in Tables 4 and 5 in detail, we notice that things are less homogeneous than for training errors. This is to be expected, as predicting the time-courses for unseen stimuli is a much harder task than predicting the timecourses for known inputs. In Table 4 the groups for which the within-group standard deviation is greater than 0.075 are highlighted in bold font. Surprisingly, there are many such groups in Hill/ANN model/system configurations. Still, in terms of the hypothesis we are testing, most groups of four do not show a pattern of decreasing validation error with increasing omega intervals. For example, the two horizontal groups of four validation errors highlighted in italic font in Table 5 illustrate two sets of validation errors that do not vary across the omega interval settings. Indeed, in some cases there is even an *increase *of error - and in other cases a slight decrease. Likewise, when we look at the vertical validation error profiles in columns (e.g. the two columns highlighted in italic font in Table 5), we notice a general pair-wise similarity for each model group. These observations confirm our hypothesis that the absolute size of the interval for *ω_ij _*is not critical. Even when data is generated with large *ω_ij _*values, the reverse-engineered models can approximate the data equally well with small and large *ω_ij _*ranges.

### Training and validation errors yeast system

Finally, we consider the *training and validation errors *we obtained from the data of the cell cycle system in Table 6. The mean training and validation errors (not shown in Table 6) for the two models obtained with the four omega intervals are presented below. *S*(*CC*) denotes the cell cycle system, and *M *(*X*) the inferred models and their underlying rate law formulations.

**- **Training: *S*(*CC*) *→ M *(*ANN*): 0.1110 *± *0.0019.

**- **Training: *S*(*CC*) *→ M *(*Hill*): 0.1116 *± *0.0018.

**- **Validation: *S*(*CC*) *→ M *(*ANN*): 0.3936 *± *0.2402.

**- **Validation: *S*(*CC*) *→ M *(*Hill*): 0.2058 *± *0.0094.

In terms of the mean training error, the two models perform almost identically. But the mean validation error of the ANN model is nearly twice that of the Hill model! This difference in predictive power is quite remarkable, even though we are testing only four omega conditions. We also observe that the variation (standard deviation) in the ANN model performance (validation error) is much higher than that of the Hill model. Clearly, the Hill rate law has more parameters and hence is more likely to fit complex curves. Still, that the ANN model mean validation error is nearly 100% higher than that of the Hill model (when the mean training errors are similar), seems to be an important observation.

We now analyze how the training and validation performance depends on the omega intervals. We observe essentially a similar pattern as in the evaluation of the synthetic systems. For the two groups of four training errors in Table 6 there seems to be hardly any variation in training error from smaller to larger omega intervals. In the four validation errors of the Hill model, we see a minor variation, but a slight rise in error as we move to larger omega intervals (if anything, the error should become smaller, as more solution possibilities are being explored). And in the validation errors of the ANN model, we notice a considerable variation in validation errors but no pattern of decrease in validation error from smaller to larger omega intervals. So overall, this seems to corroborate the results derived from the synthetic systems in Tables 2 and 3 (training errors), and Tables 4 and 5 (validation errors). It seems, that choosing large (and ad hoc) omega intervals does not make a real difference.

## Conclusions

In this study, we focused on the automated reverse-engineering (or inference) of gene-regulatory models from time-course gene expression data. The "grand challenge" in this area is to infer dynamic (time-resolved) mechanistic (quantitative cause-effect) regulatory interactions from data [4]. Currently, this task is hampered by the lack of sufficient amounts of data in terms of stimulusresponse data sets from the same system. However, as experimental techniques improve and become more affordable, more and more relevant data is likely to be produced in the future. We anticipate that multi-stimulus data on the same system is likely to reveal more of the underlying mechanistic details of GRN systems, and modeling approaches as the one presented in this study will become a part of the standard toolbox [5].

The particular focus of this study was to investigate the role of the omega parameters within a particular class of semi-mechanistic mathematical GRN model formalisms or rate laws. In ANN and Hill laws [2,13] and similar (e.g. the synergistic-system [16]) rate laws, the *ω_ij _*parameters simultaneously represent the presence or absence of transcript synthesis regulators (a discrete concept) and the strength of their regulatory influence (a continuous concept). When we reverse-engineer GRN models from time-series gene expression data, we need to define reasonable limits for these parameters, to avoid an excessively large solution search space. Often, the choice of the size of the *ω_ij _*intervals is defined in an ad hoc way or determined by trial-and-error experimentation. The hypothesis we tested in this study was that limiting *ω_ij _*to *ω_ij _*∈ [*−*1, +1] facilitates full expression without loss in accuracy of the inferred models.

To test this hypothesis, we created various data sets from three synthetic 5-gene systems (A, B, and C; see Figure 3) based on the ANN and Hill rate laws defined by Eqs. 1 and 2, and used two publicly available data sets from an 11-gene cell cycle system [11,24]. From the synthetic systems, we generated 192 training and 192 validation data sets under different omega interval conditions. We explored how the model training errors and model validation errors (predictive power) vary in relation to different settings of the omega interval. Our results suggest that the absolute size of the omega interval does not seem to have any effect on the models' predictive performance (validation error).

This result has important consequences for reverse-engineering algorithms that estimate concrete values of *ω_ij _*and other model parameters. In particular, it is not necessary to choose an excessively large interval range for *ω_ij_*. Because we need to specify a *ω_ij _*interval for all possible *n*^2 ^regulators of a GRN, large *ω_ij _*intervals have a considerable impact on the computational complexity (size of parameter solution space) of the model inference algorithm. Knowing that *ω_ij _*∈ [*−*1, +1] is sufficient is likely to improve the computing performance of such algorithms.

Clearly, more research is needed to form a more comprehensive view on the merits and limitations of GRN model inference. In particular, we need methods and tools that are capable of inferring reliable and interpretable mechanistic gene-regulatory networks from data. While empirical studies like the one presented here are important, more theoretical investigations are needed to establish how much information relating to the mechanistic gene-regulatory network structure of the underlying GRN system is actually contained in the experimental data. We need also more studies like that of Cantone and colleagues [8] that provide the basis for comprehensive studies based on real data.

## Competing interests

The authors declare that they have no competing interests.

## Authors' contributions

AM contributed to all aspects of this study, including tool implementation, experiment execution and manuscript preparation. HZ and WD contributed to the design of software and experiments and manuscript preparation. PT contributed to biological aspects of model and algorithm development.

## Declaration

This project/study was funded through the MAPPER EU-FP7 project (grant no. RI-261507). Funding for this article has come from the Biomedical Sciences Research Institute, University of Ulster, Coleraine, United Kingdom.

This article has been published as part of *BMC Systems Biology *Volume 9 Supplement 5, 2015: Selected articles from the IEE International Conference on Bioinformatics and Biomedicine (BIBM 2014): Bioinformatics. The full contents of the supplement are available online at http://www.biomedcentral.com/bmcsystbiol/supplements/9/S5.
